# Chemical Aftermath: Contamination and Cleanup Following the Tohoku Earthquake and Tsunami

**DOI:** 10.1289/ehp.119-a290

**Published:** 2011-07-01

**Authors:** Winifred A. Bird, Elizabeth Grossman

**Affiliations:** **Winifred A. Bird** is a freelance journalist living in Nagano, Japan. Her work has appeared in the *Japan Times*, *Science*, *Yale Environment 360*, *Dwell*, and other publications.; **Elizabeth Grossman**, a Portland, OR–based environmental and science writer, has written for TheAtlantic.com, *Yale Environment 360*, *Scientific American*, *The Washington Post*, and other publications. Her books include *Chasing Molecules* and *High Tech Trash*.


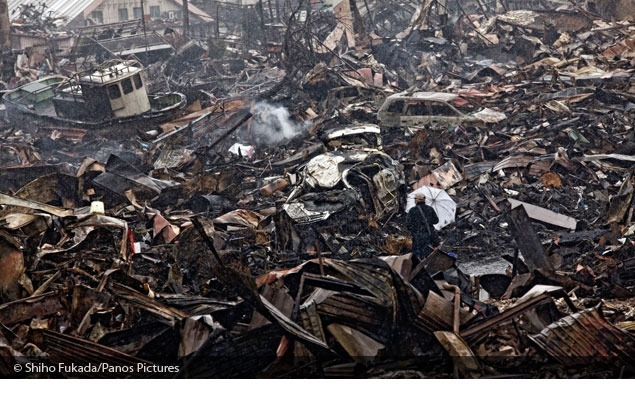


Thirty days after the most powerful earthquake and tsunami in Japan’s recorded history struck the northeastern coast of that country’s main island, the city of Ishinomaki was a scene of devastation. The busy manufacturing and industrial port town in Miyagi Prefecture,^1^ close to the epicenter of the quake, had suffered some of the worst damage of any community in the Tohoku region. Pulverized houses, skeletons of factories, and mountains of debris lined the dusty streets. Crumpled cars were tossed across graveyards, broken shipping containers strewn across fields. Ruptured oil tanks leaked glossy black liquid, bags of agrochemicals sat in iridescent puddles, and the doors to a shed labeled

“Chemical Storehouse” flapped open, revealing an emptied room. Townspeople and officials walked through this huge field of wreckage, picking at the remains of their homes or simply gazing over the surreal landscape as if immobilized by the scale of damage.^2^

**Figure:**
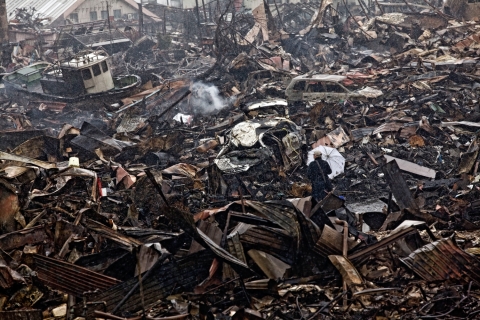
A man makes his way through the devastated town of Kesennuma, Miyagi Prefecture, March 2011. © Shiho Fukada/Panos Pictures

The magnitude 9.0 earthquake and tsunami of 11 March 2011 inundated 561 square kilometers of coastline, reaching up to 5 kilometers inland.^3^ The disaster wrought havoc from Aomori Prefecture in the north to Chiba Prefecture in the south (about 35 kilometers east of Tokyo); aftershocks affected areas far beyond the coast. The earthquake and tsunami combined may have killed nearly 23,600 people^4^ and severely damaged or destroyed more than 187,000 homes.^5^^,^^6^

Damage to the region’s industrial facilities also has been extensive. Oil refineries burst into flames in the days after the disaster, sending black smoke billowing into the air. Sewer and gas lines burst, and old electrical equipment containing polychlorinated biphenyls (PCBs) was washed away.^7^ Petro- and agrochemical plants, iron foundries, steel works, and automotive, electronics, food processing, paper, plastics, and pharmaceutical plants were among those that suffered damage.

**Figure:**
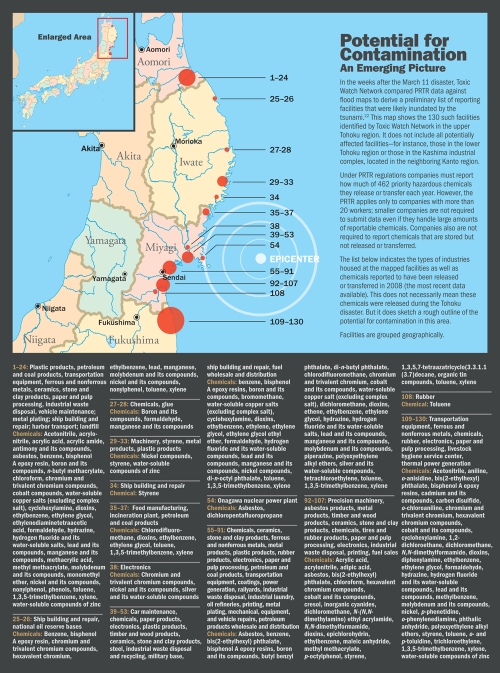
Potential for Contamination: An Emerging Picture In the weeks after the March 11 disaster, Toxic Watch Network compared PRTR data against flood maps to derive a preliminary list of reporting facilities that were likely inundated by the tsunami.^22^ This map shows the 130 such facilities identified by Toxic Watch Network in the upper Tohoku region. It does not include all potentially affected facilities—for instance, those in the lower Tohoku region or those in the Kashima industrial complex, located in the neighboring Kanto region. Download the printable copy (PDF file)

As cleanup continues in the disaster area, questions remain about the fate of chemical contaminants released by these damaged industrial facilities and other sources, and the environmental health hazards they might pose to the hundreds of thousands of people living and working in this area. Similar questions have arisen in the wake of hurricanes Katrina and Rita in 2005, the BP *Deepwater Horizon* disaster in the Gulf of Mexico in 2010, and the World Trade Center attacks on 11 September 2001. But in Japan, the vast human catastrophe and deepening Fukushima nuclear disaster have tended to eclipse these issues of chemical contamination.

## The Industries Affected

The tsunami wiped out a strip of coast supporting a wide range of land uses and industries. The Iwate coast has many fishing communities along with cement and plywood manufacturers and a large iron foundry in the badly damaged city of Kamaishi.^8^ The Miyagi coastline had an estimated 1,000 factories, including a 145,000-barrel-per-day-capacity^9^ oil refinery in Sendai, marine products processing plants all along the coast, and various manufacturing industries near the ports.^10^ Rice farms in the Sendai area—which, according to one estimate, support approximately 8% of Japan’s rice production^11^—have also been affected. The Fukushima coast has fishery-related industries, along with auto parts factories and some chemical plants.^12^

**Figure:**
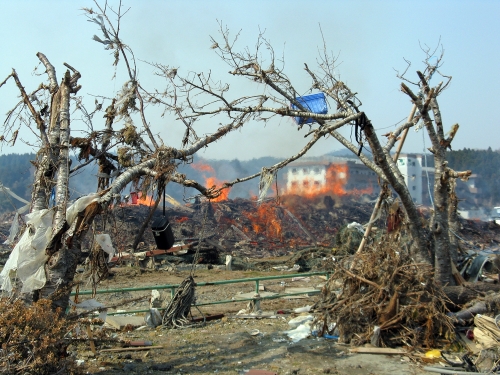
A pile of debris burns in the open in Minamisanrikucho, Miyagi Prefecture, 14 April 2011. © Winifred A. Bird

Further south, in the neighboring Kanto region, the Kashima industrial complex in Ibaraki Prefecture also suffered major earthquake and tsunami damage (although for the most part buildings remained intact).^13^ The Kashima coastal industrial zone is, according to Japanese accounts, home to the largest number of petrochemical industrial complexes in Japan.^14^ Other facilities here are a Mitsubishi Gas Chemical plant whose products include hydrogen peroxide and polycarbonates; an Adeka Company plant that produces chlorinated inorganic chemicals, flame retardants, caustic soda, and other chemicals along with oil-based food products; and a Mitsubishi Chemical plant that is Japan’s largest ethylene production site. Facilities reporting damage include the Shin-Etsu Chemical Plant, a polyvinyl chloride (PVC) factory (Shin-Etsu’s optical fiber plant in Fukushima was also damaged); Sumitomo Metal, where the earthquake toppled equipment and triggered gas tank fires; and an Asahi Glass plant that manufactures caustic soda, propylene oxide, fluorocarbon resin, and other chemicals, along with various types of flat glass used in building construction.

Dozens of high-tech and automotive production facilities also sustained damage from the quake and tsunami. Companies with plants located in Tohoku include Canon, Elpida, Fujitsu, Hitachi, Honda, Kyocera, Nissan, Panasonic, Texas Instruments (which described “substantial damage” to its semiconductor plant in Miho that affected gas, air, and chemical delivery systems), and Sony.^15^ Reports posted on company websites or noted in industry publications summarized damage very generally and primarily in terms of impact to business and production capacity. One exception is the bulletin from Freescale on its semiconductor plant in Sendai, which suffered serious damage. The company reported that when “personnel first reentered the fab [in late March] . . . they found broken ducts, pipes and windows and discovered that chemicals had leaked.”^16^ Online industry publication *Medicine Hot News* reported damage at seven major pharmaceutical companies in the Tohoku region.^17^

At the time of this writing, none of the affected major manufacturing companies had publicly reported in English the specific nature of any chemical releases related to the earthquake and tsunami. None contacted had information to share beyond what was available by way of website bulletins and similar reports compiled by industry sector publications. Disaster reports from the Japanese Ministry of the Environment,^18^ Ministry of Economy, Trade, and Industry,^19^ and Fire and Disaster Management Agency^20^ briefly described only a handful of spills including hydrochloric acid, chromium, and several unspecified hazardous materials, and damage to an ammonia tank; petrochemical spills were reported more extensively and in more detail. Here again, information beyond that in published reports was not available when requested by phone.

But data available through Japan’s Pollutant Release and Transfer Register (PRTR),^21^ comparable to the U.S. Environmental Protection Agency’s Toxics Release Inventory, indicate numerous chemicals with potential environmental and health hazards may have been present at many facilities in heavily impacted locations. In the weeks following the Tohoku disaster, Toxic Watch Network, a Tokyo-based nonprofit organization, combed the PRTR data to get a general idea of the chemicals that may have been onsite at affected facilities. The resulting list includes acrylamide, asbestos, benzene, bisphenol A, bromomethane (methyl bromide), cadmium, chromium compounds, chloroform, chlorodifluoromethane, ethylene glycol, dioxins, formaldehyde, lead, mercury, toluene, and xylene (see map, p. A292).^22^ Many of these compounds are respiratory hazards, neurotoxicants, and/or carcinogens. Many are potentially acutely toxic. Some are also environmentally persistent, which raises potential issues of long-term contamination, particularly to local soil and water.

The numerous gas and oil fires that followed the earthquake would also have released hazardous pollutants, both chemical and particulate. In addition, debris that may have included plastics, wires, vinyl products, and insulation has been burned in large, open-air piles in the town of Minamisanrikucho, Miyagi Prefecture,^23^ and possibly at other locations. Such fires have great potential to emit additional hazardous contaminants such as dioxins. These known human carcinogens result from incomplete burning of PVC, which is used extensively in wiring, construction materials, and numerous other consumer, industrial, and infrastructure applications. Dioxins can also be produced by burning seawater-soaked wood.^24^

The tsunami caused extensive damage to agricultural land and facilities in Aomori, Iwate, Miyagi, Fukushima, Ibaraki, and Chiba prefectures,^25^ where hogs and dairy and beef cattle are raised alongside crops that include rice and a variety of vegetables. Although the tsunami hit before the start of the main growing season, pesticides may have been stockpiled in agricultural locations impacted by flooding (according to the U.K.-based Agricultural Information Services consultancy, Japan is the world’s second-largest crop pesticide market after the United States, with 60% of those pesticides applied to rice ^26^). Fertilizer and feed additives could also pose potential contamination hazards to soil and surface and groundwater, and to people encountering tsunami sludge and debris. Details are not available about specific fertilizers, pesticides, and other agricultural chemicals used at farm sites affected by the tsunami.

For a city like Ishinomaki, where paper, fertilizer, feed, and chemical factories are located directly adjacent to the shore, near homes and schools, the giant wall of water destroyed conventional boundaries between “safe” and “hazardous.” No one knows if oil and chemicals spilled in one place stayed put, washed out to sea, or ended up in another part of town.

The tsunami may also have carried tsunami sludge from the bottom of bays up onto the land. “Ships come in and out of harbors, and they leak oil. There’s trash and other materials [on harbor floors],” said environmental engineer Toshiaki Yoshioka, who is a member of the Japan Society of Material Cycles and Waste Management (JSMCWM) Disaster Waste Management and Reconstruction Task Team, an academic association that has been surveying disaster waste throughout Tohoku and helping local governments develop plans to manage it. Yoshioka said the tsunami sludge could also include heavy metals, PCBs, and other pollutants washed down rivers by mines and factories before strict anti-dumping laws were passed in the late 1960s and 1970s.^27^

The problem of toxics on the harbor floors highlights the hazards that modern industrial society adds to the age-old destructive force of natural disasters. “In the past, what came up with the tsunami from the ocean was not hazardous,” Yoshioka said. “Now we are using all sorts of materials, and everything has been mixed together [by the tsunami]. If you just burn or bury [tsunami and earthquake waste] the risk to the environment is very high. We need to process the waste properly, or it will come back to haunt us.”

## Assessing the Damage

Government^28^ and independent^29^ estimates put disaster waste in the tsunami inundation area at about 25 million metric tons, and its makeup varies hugely across the disaster area. Masato Yamada, chief of the Research Center for Material Cycles and Waste Management at Japan’s National Institute for Environmental Studies (NIES), the research arm of the Ministry of the Environment, pointed out that treating all the waste as hazardous would not only be extremely expensive and time-consuming, but also would rule out the possibility of recycling some materials during the reconstruction. Yet it’s not yet clear which areas need to be treated with particular care.

“I think the tsunami sludge is probably not that dangerous except for in a few ‘hot spots’—the problem is finding them,” Yamada said. “We need to know what was in the area before the tsunami and earthquake hit. Were there industrial chemicals or agrochemicals in a certain place? That could become a hot spot.”

To an extent, that information does exist. Japan’s PRTR regulations require companies to report to local governments the quantity of 462 designated hazardous chemicals that they release into the environment or transfer to a different location each year.^30^ This information is compiled by the central government and publicly available.^31^

**Figure:**
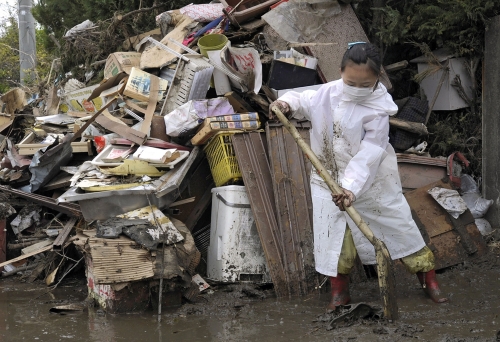
A volunteer from Tokyo cleans up a home in Higashimatsushima, Miyagi Prefecture, seven weeks after the disaster. © AP Photo/Kyodo News

But the PRTR law applies only to companies with more than 20 workers that handle certain chemicals over a specified amount; smaller companies aren’t required to submit data even if they handle large amounts of toxic chemicals.^30^ Companies are also not required to report on chemicals that are stored but not released, an information gap Kyoto University disaster planning expert Nagahisa Hirayama calls “a very big problem.” Although some of that missing information is supplied to local and central government offices under Japan’s Fire Services Act,^32^ which aims to prevent fires and limit damage from disasters including earthquakes, it is not made public in the same way that PRTR data are, according to Yoshiaki Matsuki of the Japanese Fire and Disaster Management Agency.

PRTR data—and often local disaster response plans in the United States—also miss entire categories of potential contaminants: fuels such as propane and gasoline used at factories and in vehicles; materials that are bound up in equipment and structures, including asbestos, wiring components, nonasbestos insulation, carpeting, and other flooring materials that can pose health hazards when burned; pesticides and other agrochemicals kept on farms; and chemicals kept in small quantities at homes, shops, and other nonindustrial locations. According to Toxic Watch Network director Shigeharu Nakaji, information about PRTR-listed chemicals released during the disaster will be reported to the government by June 2011 and made public early in 2012. That reporting, however, will not have informed any needed protective measures during the first weeks of cleanup.

Even existing data appeared to have barely been touched by overwhelmed scientists and government officials two months after the disaster. In May local officials in Ishinomaki and Kamaishi said they had not begun detailed investigations of damage to industrial areas or resulting chemical contamination because they were still focused on urgent relief and recovery work.^33^ By June the JSMCWM Disaster Management and Reconstruction Task Team was finalizing a strategy for identifying toxic tsunami sludge hot spots, according to Misuzu Asari, an associate professor at Kyoto University’s Environmental Preservation Research Center and a member of the task team’s sediment group. The group, which was commissioned by the Ministry of the Environment to do the work, planned to combine PRTR data with onsite soil and water tests, she said. But the situation was complicated by the fact that much debris had already been moved to collection sites. “Now we have two jobs: first, we have to identify the toxic areas, and second, we have to figure out where the debris from those areas was taken,” Asari said.

Testing for common contaminants is possible even without information about the original location of hazardous materials, and such monitoring is a growing focus in the Japanese disaster response. But environmental contaminant monitoring and testing will find only what the tests and instruments have been calibrated to seek. The siting of environmental testing in relation to potential contamination sources can also influence results and thus lead to very different decisions about resident and community health and safety, according to Scott Frickel, a Washington State University associate professor of sociology who studied contamination after Hurricane Katrina.^34^ This is an issue that has come up in the aftermath of both natural disasters like Hurricane Katrina and industrial accidents like the *Deepwater Horizon* disaster.

In Japan the ongoing recovery and related testing—which is proceeding at varying paces in different regions—falls roughly into three phases. Early on, when many disaster victims and emergency response workers are spending time surrounded by dusty debris, the tsunami sludge, air, and smoke from open-air waste burning are logical places to begin testing. (However, NIES’s Yamada pointed out that because disease-causing pathogens in the tsunami sludge are a big concern at this stage, thorough testing must be balanced with speedy cleanup.) As recovery proceeds and debris is moved from temporary to permanent storage locations, testing is needed to ensure contaminated materials are not recycled or improperly disposed of. Later, as residents begin rebuilding the worst-hit areas, redigging buried wells, fishing along the coast, and planting crops, testing for soil, surface water, and groundwater contamination will become increasingly important.

Routine monitoring of water, soil, and air quality is mainly the responsibility of prefectural governments in Japan. However, although officials in Fukushima, Miyagi, and Iwate prefectures said they were continuing routine tests wherever possible, as of the end of April they had not begun any testing specifically related to the disaster.^35^ “It’s a matter of priority,” said environmental policy expert Yoshinobu Kitamura, who is serving on a prefectural disaster waste committee in Iwate. “The first priority [for prefectural officials] is to clean stuff up and keep transport routes open. Sure, the government expects contamination, but attention to chemicals is a low priority right now.” Harried prefectural and municipal officials echoed that explanation.

**Figure:**
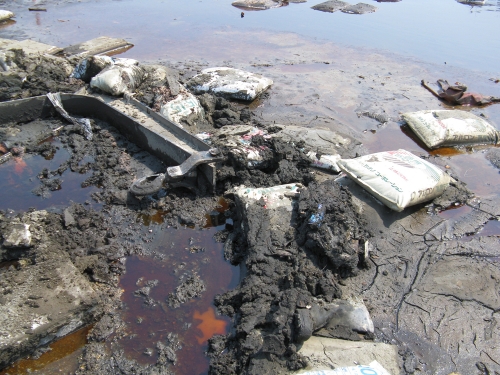
Bags of fertilizer lie strewn about the grounds of a factory in Ishinomaki, Miyagi Prefecture, 10 April 2011. © Winifred A. Bird

That situation had begun to change by June. A supplemental disaster budget approved May 2 for the Ministry of the Environment included ¥400 million (about US$5 million) for environmental monitoring aimed at assessing contamination from asbestos and hazardous materials leaked from factories and other sources.^36^ Led by the central government in consultation with local and prefectural officials, the first round of tests on soil, air, groundwater, public water areas (a legal category that includes rivers, lakes, ports, water lines, and other public water resources), and seawater and the seafloor were scheduled to wrap up by late June with results to be publicly released by early July.^37^

The Ministry had already carried out preliminary asbestos monitoring at 15 locations in three prefectures by mid-April; results fell within legally allowed limits.^38^^,^^39^ In June, asbestos testing began at approximately 130 locations including temporary houses and shelters, building demolition sites, and areas still covered in debris in Aomori, Iwate, Miyagi, Fukushima, and Chiba.^37^

But Naoki Ikeda, an Osaka lawyer with experience prosecuting soil contamination and worker health cases, warned that continued public pressure will be needed to ensure proper testing continues throughout the cleanup. Although strict environmental impact assessments are required for major public projects like dams, ports, and the construction of large garbage dumps, the same does not hold for disaster cleanup itself. “One defect of Japanese law is that even though the collection of debris is a kind of large-scale project, it’s done individually by many towns and cities. It’s seen as part of their routine responsibility even in this emergency situation,” Ikeda says. “If we lawyers and citizens don’t say anything, they may just collect the waste as usual.”

**Figure:**
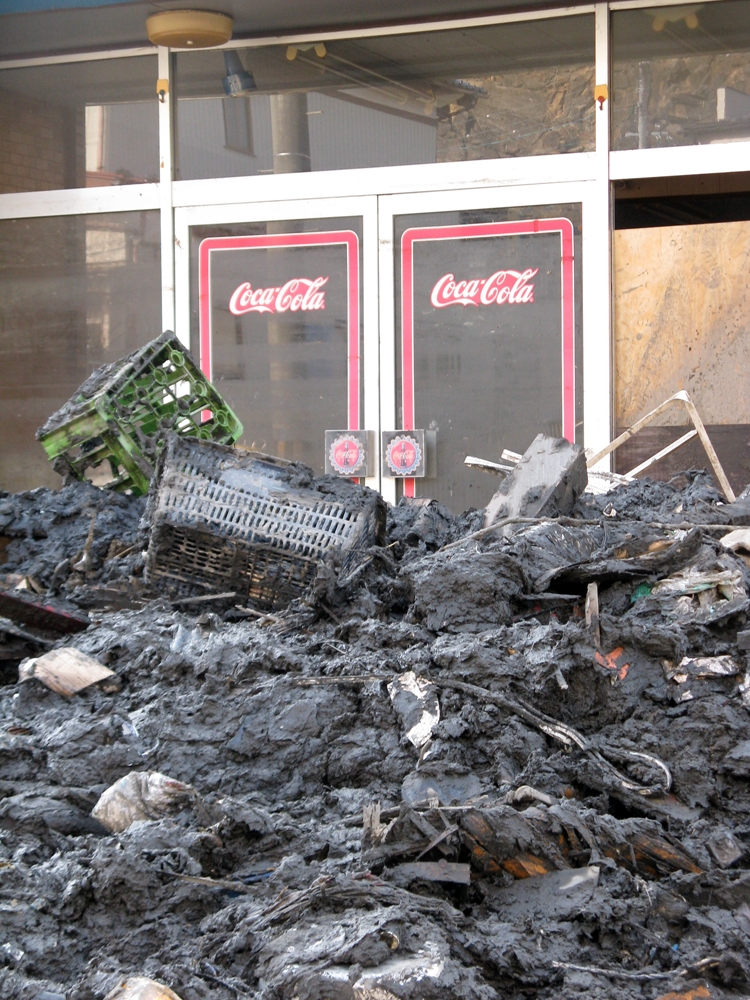
Tsunami sludge blocks the doors of a shop in Ishinomaki, Miyagi Prefecture, 10 April 2011. © Winifred A. Bird

One exception to the general lack of environmental monitoring early on was in Sendai, Miyagi’s largest city, which has legal responsibilities similar to a prefecture. In April the municipal government tested tsunami sludge samples from 32 locations, including schools, residential areas, and parks, for heavy metals, cyanide, arsenic, and PCBs.^40^ The tests turned up low levels of lead, PCBs, and arsenic at several locations—although Tetsuo Ishii, director of the city’s Environmental Management Section, said levels were similar to those detected in Sendai before the tsunami. The JSMCWM task team also tested tsunami sludge from 13 locations in Sendai for persistent organic pollutants (POPs), total petroleum hydrocarbons, *n*-hexane extractable substances, pH, and water content. All results fell below acceptable legal limits, but several samples showed high oil and POP content compared with the other samples. The researchers concluded that areas where these samples were taken may have been contaminated by damaged petrochemical factories and recommended waste from the sites be handled separately.^41^ The group plans to carry out tests on samples from across the disaster area and to broaden the tests to include heavy metals and other hazardous materials. In addition, through the end of May the Japan Environmental Measurement and Chemical Analysis Association accepted applications from people living in the disaster area to receive free soil and water quality analysis.^42^

## Protecting Residents, First Responders, and Other Cleanup Workers

Life in the disaster area, of course, has not stood still. Families are digging through the collapsed remains of their homes. Disaster victims—many of them aged and weak—are living in shelters in the midst of debris. Swarms of professional and volunteer cleanup workers and members of Japan’s military, the Self Defense Force, are scooping up tsunami sludge, clearing streets, and hauling debris to temporary disposal sites seven days a week.

Despite the lack of information about the contaminants in the dust and tsunami sludge, one month after the disaster many volunteers and contracted cleanup workers in Ishinomaki and the coastal city of Minamisanrikucho were clearing debris wearing only cotton gloves, flimsy paper masks, or no protective equipment at all. Access to damaged buildings was only partially restricted, even though aftershocks continued. It was possible to drive and walk freely through damaged industrial areas. Only a few factories were cordoned off or marked with “danger” signs.^43^

By law the professional cleanup workers and employees of damaged factories who are doing most of the cleanup work in industrial areas have a right to better protection. Japanese labor law holds employers responsible for providing proper personal protective equipment and educating workers about the risks should they fail to use it,^44^ and both industry organizations and government agencies have taken steps to make sure employers follow through.

Ayako Toyo, a media officer with the Operations Division of the National Federation of Industrial Waste Management Associations, said the 47 prefecture-level associations that make up the federation have provided safety information to companies working in the disaster area. Sugio Furuya, secretary general of the Japan Occupational Health and Safety Resource Center, said his organization—a successor to Japan’s disbanded national labor union federation—was carrying out an information campaign as well. The Ministry of Health, Labor, and Welfare also posted worker safety information in shelters and at local labor bureaus,^45^ and had distributed 90,000 masks in the disaster area by April 11^46^; unknown thousands more were distributed in later weeks. Officials from the Ministry of Labor’s Labor Standards Bureau carried out inspections of cleanup worksites in selected cities in three prefectures on April 27 and 28 to check that workers were being properly protected.^47^^,^^48^

No information has been made public about the findings of these inspections, but Hisayuki Sato, head of the Health and Safety Department at the Iwate Labour Bureau, notes that use of protective equipment has been uneven across work sites. Yuji Sakata, an official in the Ministry of Labor’s Health and Safety Planning Section, said the ministry planned to continue these inspections periodically. For its part, the Environment Ministry issued guidelines soon after the disaster hit for handling asbestos and old electrical conductors and transformers, which could contain PCBs.^19^

Health and safety guidelines for American response workers participating in cleanup and recovery efforts via the U.S. governmental or other organizations in Japan are outlined by the National Institute of Environmental Health Sciences (NIEHS) Worker Education Training Program in an online training tool titled “Controlling Hazards During the 2011 Earthquake and Tsunami Response.”^49^ Designed to walk first responders through the range of potential biological, chemical, radiation, and other hazards they may encounter, these guidelines direct workers to follow their employers’ safety and health rules, including requirements for personal protective equipment, which are mandated by the U.S. Occupational Safety and Health Administration. Comparable training manuals were developed by the NIEHS for the *Deepwater Horizon* disaster and other response efforts.

But in disasters of great geographic scope requiring large numbers of response workers, it is challenging to ensure that all workers receive adequate health and safety training and personal protective equipment, as evidenced by experiences following the *Exxon Valdez* oil spill and the *Deepwater Horizon* disaster. Judging by the number of cleanup workers observed in Ishinomaki and Minamisanrikucho without personal protective gear shortly after the earthquake, this was clearly a problem in Japan.

The thousands of volunteers on the front lines of the cleanup are in an even more vulnerable position than first responders. Ikeda, the Osaka environmental lawyer, said Japan’s strict worker health and safety laws do not protect volunteers because they are not employed by anyone. In interviews conducted in mid-April, volunteers cleaning tsunami sludge from streets and shops in Ishinomaki with Peace Boat, one of the largest nonprofit organizations working in the area, said they received scant safety training and were instructed to bring their own personal protective equipment but were not regularly reminded to use it. Simon Rogers, Peace Boat’s safety officer hired specifically for this operation, said in late April that the situation had improved greatly. By then, volunteer team leaders were receiving six hours of safety training, a safety manual had been created, and most volunteers were wearing goggles, leather gloves, and masks during their work shifts, he said. But those improvements are due to the organization’s independent efforts; no coordinated regionwide effort exists to ensure all disaster volunteers receive uniform safety training and proper equipment.^50^

Information regarding the health of residents, workers, and volunteers in the disaster area is so far scarce, aside from that related to radiation exposure. An official in the Ministry of Health, Labor, and Welfare speaking on condition of anonymity said in early June that local government offices in the disaster area did have health data, especially for people living in shelters, but that “the problem for us is how to collect that information. Everyone in the disaster area is too busy to organize and send it in [to the central government].”

The Health Sciences Division of the ministry also intended to start free health screenings targeting thousands of people in temporary housing, shelters, and damaged neighborhoods in parts of Iwate, Miyagi, and Fukushima, according to the official. However, the program has so far been plagued by difficulty. “It’s a conservative area, and many people don’t trust the central government right now. Because of decentralization, local governments are usually in charge of these kinds of health checks, so if we step in there is resistance. We’re planning to do the screening where we can get cooperation from local communities,” the official said. Because of the nuclear disaster, health checks are being carried out by Fukushima Prefecture for all citizens, including internal radiation exposure checks for people living near the damaged Fukushima Daiichi plant.^51^

Furuya said information regarding the health of cleanup workers was extremely limited as of early June. “We are monitoring the asbestos and dust situation on the ground, but health surveys of workers have not yet begun, either by us or by the Ministry of Health, Labour, and Welfare,” he said. “While no formal reports have come out yet, we’re hearing from doctors on the ground that respiratory complaints have increased among both workers and residents in the disaster area, probably because of the dust.” Nine cases of tetanus were also reported in the disaster area between March 20 and April 20, all caused by injuries sustained during the earthquake or tsunami.^52^^,^^53^

## A Global Concern

The earthquake and tsunami that hit Japan March 11 rendered meaningless many of the standard procedures used to assess, handle, and protect workers and residents from chemical hazards. Labels and signs went missing. Supervisors weren’t always available to consult. City halls and factory offices were washed away, and the need to care for thousands of homeless survivors swamped the public officials who might otherwise have focused on longer-term environmental health threats.

But is it inevitable that the health and safety challenges now facing Japan would follow a disaster that—to borrow a phrase echoed endlessly in the months after the earthquake and tsunami—“exceeded all predictions”? Or are there measures Japan and other countries can take to ensure that even in an event of this scale, residents and workers in impacted areas are protected from chemical threats?

Since the earthquake struck, Japan has come under heavy criticism for its failure to prepare for a nuclear disaster like the one at the Fukushima Daiichi power plant.^54^^,^^55^^,^^56^^,^^57^ Kyoto University disaster planning expert Hirayama said some of the same criticisms apply more broadly. “Japan had no concrete plan for dealing with chemically contaminated disaster waste before the tsunami hit,” he said. Ideally such plans would include detailed procedures for quickly assessing whether debris is hazardous or not.

Sendai’s Yoshioka added that bureaucratic sectionalism posed another man-made obstacle during the cleanup: information as well as responsibility for environmental monitoring and cleanup is divided between various ministries and branches of local governments, which increases the likelihood that, in the end, none will fulfill their shared responsibility.

**Figure:**
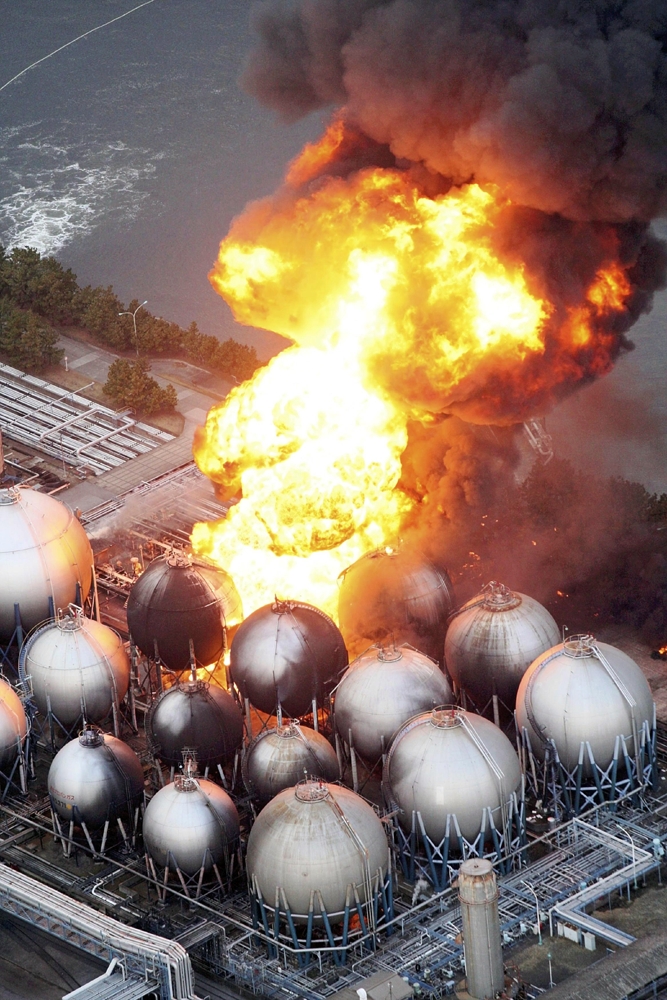
An oil refinery burns in Ichihara, Chiba Prefecture, March 2011. © AP Photo/The Yomiuri Shimbun, Kenji Tada

The problem is not Japan’s alone. In the United States detailed chemical emergency management plans are established by the Emergency Planning and Community Right-to-Know Act (EPCRA) and the Federal Emergency Management Agency (FEMA),^58^ and environmental monitoring conducted by the Environmental Protection Agency (EPA), state environmental agencies, and other federal and local government entities is often part of a federal or state government disaster response. But the question of how to address health risks posed by chemical contaminants released in the course of a disaster can easily become controversial. Disputes over where, when, and how to conduct chemical hazard assessments have arisen repeatedly in the United States—for example, during the *Exxon Valdez* cleanup in 1989, the World Trade Center cleanup after 9/11, in the aftermath of hurricanes Katrina and Rita, and during the *Deepwater Horizon* disaster and response.

Protecting emergency response workers from potential chemical and other health hazards has also been a subject of intense discussion in these events. As National Council for Occupational Safety and Health executive director Tom O’Connor points out, “Whether occupational safety and health agencies should enforce standard safety rules during an emergency or whether they should operate in a ‘non-enforcement mode’ has been a hotly debated topic in the U.S.” One example involves the shortened hazardous waste operations (HAZWOPER) training that was instituted for certain emergency workers during the *Exxon Valdez* oil spill response^59^ to facilitate rapid mobilization of a large oil spill cleanup work force. But during the *Deepwater Horizon* response, intense discussion revolved around whether the shorter courses provide adequate worker training and protection.

Although certain reporting about the use and storage of hazardous chemicals is legally required by EPCRA and by local government emergency planning programs, the reporting requirements themselves resulted from a political process and do not include all hazardous materials. Similarly, decisions about what information is made public and what tests are conducted are often subject to political negotiations. Therefore, what is considered politically or, indeed, logistically feasible can take precedence over what may be ideal in terms of health protections.

Assessments of potential chemical health hazards resulting from disasters also are affected by how much is known about predisaster environmental conditions and local levels of pollution. How such conditions are taken into consideration inevitably influences what is considered “normal” or “safe” for residents and workers in the affected area.^60^

But again, health and safety are not the sole considerations in these assessments; confidential business and security considerations, practicality, cost, and the desire to return to business as usual all come into play. All these complications prompt the question of whether more emphasis should be placed on the kind of upstream chemical pollution prevention and hazard elimination that can be achieved through green chemistry.^61^ A transition to more environmentally benign materials and manufacturing processes could help protect community, environmental, and emergency worker health and safety even when natural disasters exceed our worst predictions.

In the course of reporting this article, we contacted federal agencies, including the Centers for Disease Control and Prevention, EPA, FEMA, and NIEHS in the United States, and a number of corresponding agencies in Japan, to ask how emergency management plans for chemical hazards have worked in the course of actual disasters and how assessment of such potential hazards have been evaluated in the immediate aftermath of disasters. These agencies directed us to the copious—but general—information available online that describes existing chemical emergency management plans and regulations. But many open questions remain about the implementation and adequacy of these policies, particularly in the event of a disaster with such wide-ranging potential health hazards as the Tohoku earthquake and tsunami.

The situation in Japan is evolving, and it’s clear that in an event like the March 11 disaster, primary concerns will always be the immediate safety and recovery for everyone affected. But even during initial rescue efforts, responders need to be protected against chemical hazards, and when cleanup and rebuilding efforts begin, the potential health hazards posed by chemical contaminants become increasingly important. Judging from the extreme difficulty of obtaining concrete, detailed information about potential chemical hazards following the Japan disaster, this appears to be an aspect of emergency preparedness that, despite well-established formal disaster-response plans, remains inadequately addressed.
